# Kidney Allograft Rejection as an Independent Nontraditional Risk Factor for Post-Transplant Cardiovascular Events

**DOI:** 10.34067/KID.0000000773

**Published:** 2025-03-19

**Authors:** Peemai Amornkanjanawat, Stephen J. Kerr, Thunyatorn Wuttiputhanun, Natavudh Townamchai, Asada Leelahavanichkul, Pichaya Tantiyavarong, Kearkiat Praditpornsilpa, Somchai Eiam-Ong, Yingyos Avihingsanon, Suwasin Udomkarnjananun

**Affiliations:** 1 Division of Nephrology, Department of Medicine, Faculty of Medicine, Chulalongkorn University and King Chulalongkorn Memorial Hospital, The Thai Red Cross Society, Bangkok, Thailand; 2 Biostatistics Excellence Center, Research Affairs, Faculty of Medicine, Chulalongkorn University, Bangkok, Thailand; 3 HIV-NAT, The Thai Red Cross AIDS Research Center, Bangkok, Thailand; 4 The Kirby Institute, University of New South Wales, Sydney, New South Wales, Australia; 5 Excellence Center for Organ Transplantation (ECOT), King Chulalongkorn Memorial Hospital, The Thai Red Cross Society, Bangkok, Thailand; 6 Renal Immunology and Renal Transplant Center of Excellence, Faculty of Medicine, Chulalongkorn University, Bangkok, Thailand; 7 Thai Transplantation Society, Bangkok, Thailand; 8 Center of Excellence on Translational Research in Inflammation and Immunology (CETRII), Department of Microbiology, Faculty of Medicine, Chulalongkorn University, Bangkok, Thailand; 9 Immunology Unit, Department of Microbiology, Chulalongkorn University, Bangkok, Thailand; 10 Department of Clinical Epidemiology, Faculty of Medicine, Thammasat University, Pathum Thani, Thailand

**Keywords:** acute allograft rejection, acute rejection, cardiovascular events, heart failure, kidney transplantation, transplant outcomes

## Abstract

**Key Points:**

- Kidney allograft rejection is an independent risk factor for post-transplant cardiovascular events (CVEs), regardless of kidney allograft function.
- Time-updated post-transplant variables were more associated with post-kidney transplantation CVEs than using the pretransplant variables only.
- Proper screening protocol for high-risk recipients may be necessary to reduce the incidence of post-kidney transplantation CVEs.

**Background:**

Cardiovascular death is the leading cause of mortality in kidney transplant recipients (KTRs). Although risk factors for post-transplant cardiovascular events (CVEs) have been established, previous studies primarily focused on factors at the time of transplantation without integrating post-transplant factors into the analyses. In addition, most studies were conducted in a mixed population of cyclosporine A and tacrolimus-based immunosuppression, which have different metabolic effects. This study aims to evaluate factors for post-transplant CVEs, including both pretransplant and post-transplant variables, specifically in a population of KTRs receiving tacrolimus-based immunosuppression.

**Methods:**

Competing risk regression was performed modeling participant demographics, transplant characteristics, and post-transplant time-updated variables. The primary outcome was the composite of post-transplant CVEs, which included myocardial infarction, heart failure, ischemic stroke, peripheral arterial disease, and cardiovascular death.

**Results:**

The incidence of post-transplant CVEs was 15.88 per 1000 patient-years among 553 KTRs included in the study. Key factors significantly associated with post-transplant CVEs included recipient age, diabetes mellitus status, post-transplant hemoglobin A1c, 24-hour urine creatinine clearance, post-transplant serum calcium, and rejection. KTRs with a history of T-cell–mediated rejection or antibody-mediated rejection were at a three-fold (95% confidence interval, 1.22 to 7.37; *P* value 0.016) and 3.38-fold (95% confidence interval, 1.13 to 10.09; *P* value 0.029) higher risk for post-transplant CVEs, respectively. Compared with models using pretransplant factors alone, models that included both pretransplant and post-transplant variables demonstrated significantly higher prediction performance.

**Conclusions:**

Allograft rejections significantly increased the risk of post-transplant CVEs. Surveillance protocols for post-transplant CVEs should include KTRs with a history of allograft rejection, in addition to the traditional high-risk groups.

## Visual Abstract

**Figure:**
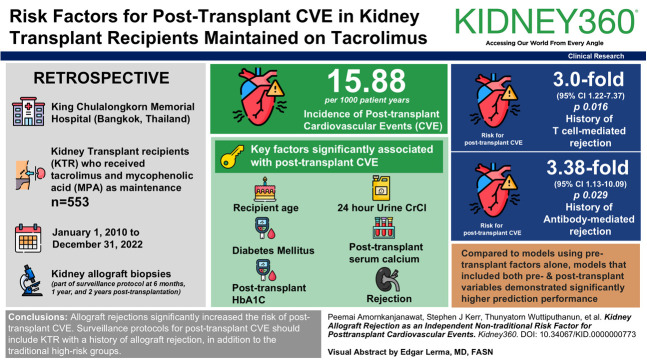

## Introduction

Cardiovascular complications are the leading cause of death in ESKD patients, with an incidence of 2000–3000 cardiovascular deaths per 100,000 person-years.^1^ Kidney transplantation significantly reduces this risk, lowering the incidence to approximately 500–1000 deaths per 100,000 person-years.^1^ Transplant recipients experience fewer cardiac events, including heart failure and ischemic heart disease, than dialysis patients,^2^ although rates remain higher than the general population.^3–5^

Traditional and nontraditional risk factors for post-transplant cardiovascular disease are well-established.^5–8^ Nontraditional risks specific to patients with CKD, including kidney transplant recipients (KTRs), encompass kidney (allograft) dysfunction, proteinuria, CKD-mineral and bone disorder, anemia, and immunosuppressive medications.^5–10^ Inflammation has been proposed as one of the nontraditional risk factors for post-transplant cardiovascular complications.^11–13^

Allograft rejection is associated with inflammation, leading to atherosclerosis or arteriolosclerosis in both animal models and clinical studies.^13–19^ Unlike cardiac transplant recipients, who directly experience coronary artery disease because of cardiac allograft rejection and arteriolosclerosis of the coronary arteries,^20^ evidence linking kidney transplant rejection to post-transplant cardiovascular events (CVEs), independent of allograft dysfunction and proteinuria, is limited. In addition, previous studies were conducted during the cyclosporine A era,^16–19,21^ before advancements in immunosuppression, HLA typing and matching, donor-specific antibody (DSA) testing, and improved allograft rejection classification, all of which have since reduced both rejection and CVEs.^22–26^ Moreover, previous studies focused on pretransplant variables, neglecting dynamic post-transplant factors, potentially overestimating pretransplant risks.

This study aimed to determine whether kidney allograft rejection contributes to post-transplant CVEs, independent of allograft function or proteinuria. The incidence and risk factors for CVEs after kidney transplantation were examined in the current era, with tacrolimus as the primary immunosuppressive medication. Cox and competing risk regression, using time-varying post-transplant variables, were applied to estimate cause-specific hazards, subhazards, and predicted risks.^27^

## Methods

### Study Design and Population

This is a retrospective cohort study that included KTRs who received transplantation at King Chulalongkorn Memorial Hospital, Bangkok, Thailand. Every transplant recipient from January 1, 2010, to December 31, 2022, was included, excluding those younger than 18 years on the transplant day. The year 2010 was chosen as the starting point because it marked the broad use of tacrolimus in Thailand and the establishment of a proper organ allocation system.^28^ Only KTRs who received tacrolimus and mycophenolic acid (MPA) as their maintenance regimen were included in the study. This approach was taken to examine the association between the standard immunosuppressive regimen used during the study period and cardiovascular outcomes, as well as to enhance the homogeneity of the study population with respect to immunosuppression. Tacrolimus predose concentration (C_0_) was targeted at 7–12 ng/ml during the first 3 months and then reduced to 5–8 ng/ml thereafter. Mycophenolate mofetil (or equivalent dose of mycophenolate sodium) was prescribed at 1000–1500 mg/d. In addition to antithymocyte globulin or basiliximab, methylprednisolone was used for induction at 500 mg/d for 3 days, followed by a switch to prednisolone at 1 mg/kg per day, which was tapered to a maintenance dose of 5 mg/d. KTRs who were lost to follow-up, had missing post-transplant follow-up data (as described in the subsequent section) for more than 1 year, or had a follow-up period of <30 days after transplantation were excluded from the analyses. Those diagnosed with primary nonfunction of the kidney allograft were excluded. All KTRs were prescribed statins starting 1 month after transplantation, regardless of their LDL level, as per the protocol in our transplant center.

Kidney allograft biopsies were performed as part of a surveillance protocol at 6 months, 1 year, and 2 years post-transplantation. For-cause biopsies were conducted in cases of unexplained kidney allograft dysfunction or proteinuria. DSA testing was not routinely included in the surveillance protocol, but was typically performed when allograft dysfunction occurred. KTRs with positive DSA results underwent allograft biopsy. Cytomegalovirus (CMV) prevention primarily followed a preemptive strategy, except for CMV D+/R− KTRs, who received 3–6 months of valganciclovir prophylaxis.

This study was approved by the Institutional Review Board of the Faculty of Medicine, Chulalongkorn University, Bangkok, Thailand, in compliance with international guidelines for human research protection, including the Declaration of Helsinki, the Belmont Report, the Council for International Organizations of Medical Sciences Guidelines, and the International Conference on Harmonization in Good Clinical Practice (IRB No. 0259/66). The data used for the analyses were decrypted to ensure anonymity, guaranteeing that no participants could be identified. The clinical and research activities being reported are consistent with the Principles of the Declaration of Istanbul, as outlined in the Declaration of Istanbul on Organ Trafficking and Transplant Tourism.

### Data Extraction and Outcomes

All clinical data and post-transplant laboratory investigations standard for kidney transplantation care were included in the analyses. Pretransplant data for donors and recipients, along with transplant-related data such as ABO blood group compatibility, HLA mismatch, parel reactive antibody, CMV serostatus, ischemic time, immunosuppression, and delayed graft function incidence, were incorporated as variables in the model. During the post-transplant period, time-varying variables recorded at each follow-up visit were used in the analyses, including post-transplant diabetes, T-cell–mediated rejection (TCMR), antibody-mediated rejection (ABMR), BP, hemoglobin, electrolytes, creatinine, uric acid, albumin, HDL, LDL, fasting glucose, hemoglobin A1c (HbA1c), parathyroid hormone, 24-hour urine protein, creatinine clearance, and tacrolimus concentrations. The diagnosis of TCMR and ABMR was based on the Banff classification 2019.^26^ TCMR includes acute TCMR and chronic active TCMR but not borderline change. ABMR includes active and chronic active ABMR but not chronic (inactive) ABMR. TCMR and ABMR were recorded separately; thus, KTRs exhibiting both types of rejection were classified as having both TCMR and ABMR, rather than being categorized as a separate mixed rejection group.

The primary outcome was the incidence of a composite end point of postkidney transplant CVEs, including cardiovascular death, myocardial ischemia or infarction, heart failure, peripheral arterial disease, and ischemic stroke. The definition of each outcome is presented in Supplemental Table 1.^29–31^

### Statistical Analyses

The incidence of post-transplant CVEs was calculated and presented as an incidence rate per 1000 patient-years. To investigate the risk factors for post-transplant CVEs, competing risk regression was performed with noncardiovascular deaths and graft loss defined as competing events.^27,32^ Post-transplant variables that had multiple values for multiple follow-up visits were analyzed as time-updated covariates selecting the value closest to post-transplant visits at 6 months, 1 year, and every year until the last follow-up year. Study participants who did not experience a CVE were censored at their most recent follow-up visit. All variables, including post-transplant follow-up laboratory investigations, were first analyzed using univariable competing risk regression models. Covariates with a subhazard ratio (SHR) *P* value of <0.1 in the univariable analysis were selected for inclusion in the multivariable model. Multicollinearity among variables included in the multivariable competing risks regression model was assessed using the variance-covariance matrix. The final multivariable model was derived using stepwise backward elimination and retaining variables, which minimized Akaike's information criterion. Bootstrap resampling using 1000 replications was performed for internal validation.

Based on the final multivariable model, a more simplified, easy-to-use scoring system was calculated to allow clinicians to approximately estimate the overall risk of post-transplant CVEs. These scores were developed on the basis of the *β*-coefficients of each variable in the model. We calculated the time updated area under the receiver operating characteristics curve for the cause-specific hazard functions of these models and a calibration plot to show relevance to the original model. All statistical analyses were performed in Stata 17.0 (StataCorp LLC, College Station, TX).

## Results

### Baseline Characteristics, Incidences, and Outcomes of Post-Transplant CVEs

**Figure 1:**
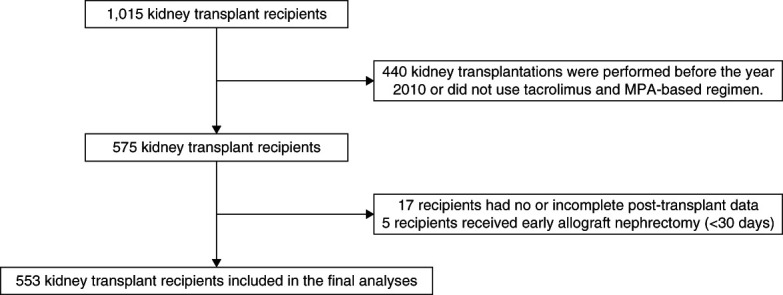
**Study flow diagram.** MPA, mycophenolic acid.

Figure 1 shows the flow diagram of the study. Out of a total of 1015 KTRs at our center, 575 received a transplant after 2010 and were on a tacrolimus and MPA-based regimen. After excluding KTRs with missing data or those who underwent allograft nephrectomy within 30 days, 553 KTRs were included in the final analysis. Baseline characteristics of KTRs are presented in Table 1. The age at transplantation was 44.1±11.9 years, with 58% being male recipients. Most of the KTRs (87.0%) received hemodialysis, whereas 8.5% were on peritoneal dialysis before transplantation, with a median dialysis vintage of 3.5 years (interquartile range [IQR], 1.9–5.9 years). Deceased donors accounted for 56.8% of transplants, with most considered low-to-moderate immunologic risk (HLA mismatch 2.8±1.5, median parel reactive antibody 0 [0–0]). Statins were prescribed initially to all 553 KTRs. Among them, 43 KTRs had a history of switching statin therapy (*e.g*., from atorvastatin to pravastatin) and 9 KTRs had statins completely stopped. Of the nine KTRs who discontinued statin therapy, one developed a myocardial infarction (11%), a rate comparable with KTRs who continued statin therapy (15% or 80 composite events of 544 KTRs).

**Table 1:** Baseline characteristics of the included kidney transplant recipients

Variable	Total Cohort (*n*=553)
**Recipient characteristics**	
Recipient age^a^, yr, mean±SD	44.1±11.9
Recipient male sex, *n* (%)	322 (58)
Recipient height, cm, mean±SD	163.7±8.6
Body weight, kg, mean±SD	59.3±12.3
Cause of kidney disease, *n* (%)	
*Diabetic nephropathy*	68 (12.3)
*Hypertensive nephropathy*	11 (2.0)
*Glomerular disease*	148 (26.8)
*Cystic kidney disease*	28 (5.0)
*Others^b^*	12 (2.2)
*Unknown^c^*	286 (51.7)
Mode of dialysis, *n* (%)	
*Preemptive transplantation*	25 (4.5)
*Hemodialysis*	481 (87.0)
*Peritoneal dialysis*	47 (8.5)
Dialysis vintage, yr, median (IQR)	3.5 (1.9–5.9)
Repeated kidney transplantation, *n* (%)	18 (3.2)
Comorbidities, *n* (%)	
*Hypertension*	491 (88.8)
*Dyslipidemia*	156 (28.2)
*Diabetic mellitus*	87 (15.7)
*Ischemic heart disease*	22 (4)
*History of heart failure*	15 (2.7)
*Peripheral artery disease*	4 (0.7)
*History of cerebrovascular disease*	17 (3)
*History of malignancy*	80 (14.5)
History of smoking, *n* (%)	74 (13.4)
History of alcohol drinking, *n* (%)	54 (9.7)
Medications, *n* (%)	
*Aspirin*	56 (10.1)
*Clopidogrel*	8 (1.4)
History of parathyroidectomy, *n* (%)	38 (6.8)
**Donor characteristics**	
Donor age, yr, mean±SD	38±11.8
Donor male sex, *n* (%)	355 (64.2)
Types of donors, *n* (%)	
*Deceased donor*	314 (56.8)
*Living related donor*	189 (34.2)
*Living unrelated donor*	50 (9)
Cause of death, *n* (%)	
*Traumatic brain injury*	207 (37.4)
*Cardiovascular disease^d^*	95 (17.2)
*Cardiac arrhythmia*	7 (1.7)
*Central nervous system tumor*	4 (0.7)
Donor creatinine, mg/dl, mean±SD	1.29±1.05
Donor hypertension, *n* (%)	34 (6.1)
Donor diabetic mellitus, *n* (%)	5 (1)
**Transplant characteristics**	
ABO incompatibility, *n* (%)	37 (6.7)
Total ischemic time, min, median (IQR)	754 (58–1131)
HLA MM, mean±SD	2.8±1.5
PRA, %, median (IQR)	0 (0–0) (range 0%–98%)
Multi-organ transplantations, *n* (%)	11 (2)
CMV serostatus, *n* (%)	
*CMV IgG D+/R+*	542 (98)
*CMV IgG D+/R−*	10 (2)
*CMV IgG D−/R−*	1 (0.2)
Induction therapy, *n* (%)	
*No induction*	3 (0.5)
*Antithymocyte globulin*	63 (11.4)
*IL-2 receptor antagonists*	487 (88.1)
DGF^e^, *n* (%)	99 (17.9)

CMV, cytomegalovirus; DGF, delayed graft function; IQR, interquartile range; MM, mismatch; PRA, parel reactive antibody.

a Age at transplantation.

b Other causes of kidney diseases included kidney stone, obstructive uropathy, analgesic nephropathy, and contrast-induced AKI.

c Unknown cause was defined if patients had no clinical clues for the etiology of kidney disease and no kidney biopsy was done.

d Cardiovascular disease included myocardial ischemia/infarction and hemorrhagic/ischemic stroke.

e Delayed graft function was defined as the need for dialysis during the first week after transplantation.

Table 2 presents the incidence of post-transplant CVEs. The median follow-up time was 6.15 (3.70–9.00) years. The incidence of myocardial infarction or ischemia was 7.16 (95% confidence interval [CI], 4.84 to 10.59), heart failure was 5.41 (95% CI, 3.45 to 8.48), peripheral arterial disease was 4.54 (95% CI, 2.78 to 7.42), and ischemic stroke was 3.40 (95% CI, 1.93 to 5.99) per 1000 patient-years. The incidence of cardiovascular death was 2.52 (95% CI, 1.31 to 4.85) per 1000 patient-years. Overall, the incidence of composite events (myocardial infarction or ischemia, heart failure, peripheral arterial disease, ischemic stroke, and cardiovascular death) was 15.88 (95% CI, 12.17 to 20.74) per 1000 patient-years (81 events in 553 KTRs).

**Table 2:** Follow-up time and incidences of the study's outcomes

Overall follow-up time, yr, median (IQR)	6.15 (3.70–9.00)
Time to composite outcome, yr, median (IQR)	3.34 (1.11–6.11)
Incidence rate of composite outcome, per 1000 patient-years (95% CI)	15.88 (12.17 to 20.74)
All-cause mortality, per 1000 patient-years (95% CI)	8.98 (6.35 to 12.69)
**Events, per 1000 patient-years (95% CI)**	
Myocardial infarction or ischemia	7.16 (4.84 to 10.59)
Heart failure	5.41 (3.45 to 8.48)
Peripheral arterial disease	4.54 (2.78 to 7.42)
Ischemic stroke	3.40 (1.93 to 5.99)
Death from cardiovascular disease	2.52 (1.31 to 4.85)

CI, confidence interval; IQR, interquartile range.

We performed a secondary analysis on the basis the median time to composite outcome to explore the natural history of each event. At the median time to composite outcome (3 years post-transplantation), the mean tacrolimus C_0_ was 4.7±2.5 ng/ml (median 4.9 ng/ml [IQR, 3.3–6.0]), the mean MPA dose (equivalent to mycophenolate mofetil) was 922.5±460 mg/d (median 1000 mg [IQR, 1000–1000]), and the mean prednisolone dose was 3.9±1.7 mg/d (median 5 mg [IQR, 2.5–5.0]). Heart failure, peripheral arterial disease, ischemic stroke, and the composite outcome occurred more frequently before 3 years. By contrast, myocardial infarction/ischemia, cardiovascular death, and all-cause mortality had higher incidence rates after 3 years (Supplemental Table 2).

The mortality risk of KTRs who developed post-transplant CVEs is illustrated in Supplemental Figure 1. Competing risk regression showed adjusted SHR of post-transplant CVEs for overall mortality of 5.80 (95% CI, 2.36 to 14.25).

### Competing Risk Regression for Factors Predicting Post-Transplant CVEs

The results of the univariable competing risk regression are presented in Supplemental Table 3. The incidence of diabetes mellitus newly diagnosed post-transplant was 16.20 (95% CI, 12.38 to 21.21) per 1000 patient-years, with a median time of diagnosis of 0.64 (0.31 to 2.76) years after transplantation. The incidence of TCMR and ABMR was 28.90 (95% CI, 23.50 to 35.53) and 20.65 (95% CI, 16.25 to 26.24) per 1000 patient-years, with a median time to first occurrence of 0.70 (0.33 to 1.90) and 0.85 (0.11 to 4.08) years after transplantation, respectively. Supplemental Figure 2 presents the mean and SD of laboratory parameters after kidney transplantation.

**Figure 2:**
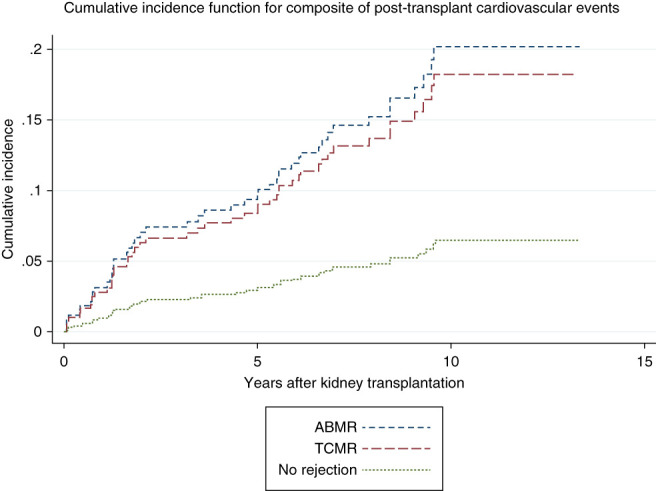
**Cumulative incidence function for composite of post-transplant CVEs (ischemic heart disease, heart failure, peripheral vascular disease, ischemic stroke, and cardiovascular death) based on of kidney allograft rejection status.** ABMR, antibody-mediated rejection; CVE, cardiovascular event; TCMR, T-cell–mediated rejection.

The final multivariable models based on backward elimination criteria are presented in Table 3. Model 1 included both pretransplant and post-transplant variables: recipient age, pretransplant diabetes mellitus, diabetes mellitus newly diagnosed post-transplantation, TCMR, ABMR, post-transplant serum calcium concentrations, and 24-hour urine creatinine clearance. Bootstrapping for internal validation of the model is presented in Supplemental Table 4, demonstrating comparable estimates to the original model. TCMR (SHR, 3.00; 95% CI, 1.22 to 7.37) and ABMR (SHR, 3.38; 95% CI, 1.13 to 10.09) were independent risk factors for the composite of post-transplant CVEs. The cumulative incidence function of post-transplant CVEs based on TCMR and ABMR status compared with KTRs without rejection is illustrated in Figure 2. Model 2 used only pretransplant variables for predicting post-transplant CVEs and included recipient age, body mass index, deceased donor transplantation, and pretransplant diabetes mellitus status. Harrell's C-statistic for model 1 was significantly better than model 2, with a difference in C-statistic between the models of 0.05 (95% CI, 0.01 to 0.09). An exploratory analysis comparing the incidence of CVEs between KTRs with and without rejection is presented in Supplemental Table 5.

**Table 3:** Final multivariable models for the prediction of post-transplant cardiovascular events based on the Akaike information criterion

Variables	Model 1: Multivariable Analysis Includes Pretransplant and Post-transplant Variables			Model 2: Multivariable Analysis Includes Only Pretransplantation Variables		
	SHR	95% CI	*P* Value	SHR	95% CI	*P* value
Recipient age	1.05	1.02 to 1.08	0.002	1.04	1.02 to 1.07	0.001
Body mass index	—	—	—	1.08	1.01 to 1.16	0.029
Deceased donor transplantation	—	—	—	1.72	0.95 to 3.12	0.072
Pretransplant recipient diabetes mellitus	6.08	2.87 to 12.90	<0.001	4.80	2.58 to 8.96	<0.001
Newly diagnosed post-transplant diabetes mellitus^a^	2.70	1.06 to 6.84	0.036	—	—	—
Previous T-cell–mediated rejection^a^	3.00	1.22 to 7.37	0.016	—	—	—
Previous ABMR^a^	3.38	1.13 to 10.09	0.029	—	—	—
Post-transplant calcium concentrations^a^	0.59	0.37 to 0.94	0.028	—	—	—
Post-transplant HbA1c^a^	1.16	0.98 to 1.36	0.077	—	—	—
24-h urine creatinine clearance^a^	0.98	0.97 to 0.99	<0.001	—	—	—

C-statistic for model 1: 0.90 (95% CI, 0.87 to 0.94). C-statistic for model 2: 0.86 (95% CI, 0.80 to 0.91). Difference of C-statistic between model 1 and 2: 0.05 (95% CI, 0.01 to 0.09), *P* value 5 0.038. ABMR, antibody-mediated rejection; CI, confidence interval; HbA1c, hemoglobin A1c; SHR; subhazard ratio.

a Time-varying variables during the post-transplant follow-up.

The mean prednisolone dose for rejection treatment, administered immediately on diagnosis, was 26.4±10.9 mg/d, significantly higher than the standard maintenance dose of 5 mg/d per protocol. Our rejection treatment protocol includes intravenous methylprednisolone 500 mg/d for 3 days, followed by 0.5 mg/kg per day of prednisolone for TCMR and plasmapheresis with intravenous immunoglobulin (±rituximab) for ABMR. Although corticosteroid dose was significant in the univariable analysis, it exhibited multicollinearity with rejection in the multivariable model for predicting composite CVEs, complicating the interpretation of the independent effects of rejection treatments. In the multivariable model, corticosteroid dose had a less significant *P* value (SHR, 1.02; 95% CI, 0.99 to 1.04; *P* value 0.064) compared with rejection. To optimize model fit and reduce collinearity, corticosteroid dose was removed from the final multivariable model based on backward elimination criteria (Akaike's information criterion) and its weaker significance, while rejection remained a significant predictor of composite CVEs.

Secondary analyses exploring the factors predicting each individual post-transplant CVE are presented in Supplemental Table 6, using a methodology similar to that of the composite outcome.

### Simplified Score to Predict Post-Transplant CVEs

**Figure 3:**
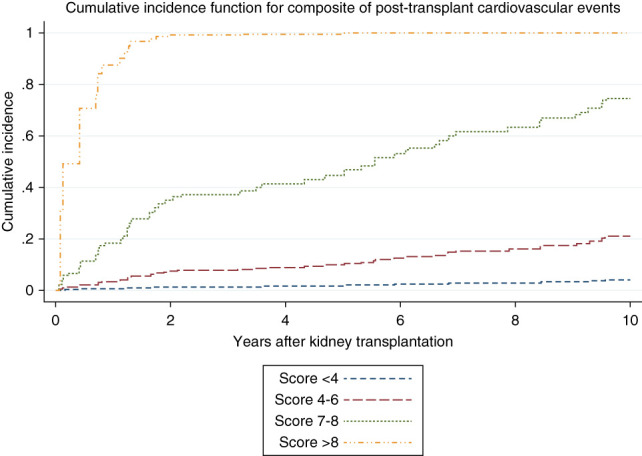
Cumulative incidence function according to the simplified risk score on post-transplant CVEs.

Based on the multivariable competing risk regression model 1, a simplified scoring was developed from the coefficient of each variable (Supplemental Table 7). Continuous variables were categorized based on linearity assessments and clinical relevance. Recipient age was categorized with a cutoff at 40 years, determined by the linearity assessment in the model (Supplemental Figure 3). The final score to predict post-transplant CVEs can classify the risks as per score categories presented in Table 4. The calibration plot of the simplified score on the observed against expected probabilities is presented in Supplemental Figure 4, showing the 95% CI of the expected prediction probabilities within the reference range. We found no significant difference in the time updated area under the receiver operating characteristics curve for the cause-specific hazard between the simplified and original model (difference in C-statistic, 0.02; 95% CI, −0.01 to 0.06; *P* value 0.168). Figure 3 presents the cumulative incidence function on the basis of the simplified risk score on post-transplant CVEs.

**Table 4:** Simplified score for the prediction of postkidney transplantation cardiovascular events

Simplified Variables	Simplified Score
Recipient age ≥40 yr at transplantation	3 points
Pretransplant recipient diabetes mellitus OR	2 points OR
Newly diagnosed post-transplant diabetes mellitus	1 point
Previous T-cell–mediated rejection	1 point
Previous ABMR	2 points
Post-transplant calcium concentrations <8.5 mg/dl	1 point
Post-transplant HbA1c ≥7	1 point
24-h urine creatinine clearance <60 ml/min	1 point

Final Score (Max 11)		
Final Score to Predict 5-yr Post-Transplant CVEs and Their Corresponding Risks		
Risk Score	Cumulative Incidence Rate of Post-Transplant CVEs with 95% CI	Interpretation
<4	0.63 (0.09 to 4.43) %	<5% (low risk)
4–6	9.76 (5.86 to 16.01) %	5%–20% (moderate risk)
7–8	51.66 (31.15 to 75.73) %	30%–80% (high risk)
>8	100 (100 to 100) %	>80% (very high risk)

C-statistic for simplified score: 0.88 (95% CI, 0.83 to 0.93). Difference of C-statistic between original model and simplified score: 0.02 (95% CI, 20.01 to 0.06), *P* value 5 0.168. ABMR, antibody-mediated rejection; CI, confidence interval; CVE, cardiovascular event; HbA1c, hemoglobin A1c.

## Discussion

This study provides important information on the risk of post-transplant CVEs, particularly the consequences of kidney allograft rejection in contemporary transplant practice and immunosuppression regimens. These risks were independent of allograft function or proteinuria. The simplified scoring system developed in this study allows for easier use in clinical practice and demonstrated comparable performance to the original prediction model.

In KTRs, the use of immunosuppressive medications is a key factor contributing to post-transplant CVEs.^33^ Cyclosporine A is known for its dyslipidemia and hypertension side effects, while tacrolimus has pancreatic islet cell toxicity, resulting in post-transplant diabetes mellitus or worsening preexisting diabetes mellitus.^34–36^ Serum LDL and BP did not significantly affect post-transplant CVEs in our multivariable analysis, which might partly be explained by the absence of cyclosporine A in the immunosuppression of the included population. Our post-transplant care protocol mandates statins for all KTRs starting 1 month after transplantation. Consequently, comparing outcomes between KTRs with and without statin therapy was not feasible in this study. Statin use was widely accepted within this cohort, with only a few KTRs experiencing myalgia or mild myositis. These side effects resolved after brief discontinuation and were generally well-tolerated upon rechallenge with an alternative statin. Statins are well-established for reducing CVEs in KTRs and are recommended for all KTRs, regardless of LDL levels, as endorsed by the Kidney Disease Improving Global Outcomes guidelines.^37–41^ The findings of our study should be interpreted within the framework of this universal statin protocol.

Ischemic stroke and peripheral arterial disease were more common within the first 3 years post-transplant, whereas myocardial infarction, cardiovascular death, and all-cause mortality occurred more frequently after 3 years. The reasons for the differing timing of events remain unclear. However, because these CVEs are common among both ESKD patients and KTRs, they could represent either preexisting conditions at the time of transplant or *de novo* events occurring after transplantation. Immunosuppressive agents—such as cyclosporine, tacrolimus, mammalian target of rapamycin inhibitors, and corticosteroids—are known to induce metabolic abnormalities, including dyslipidemia, diabetes mellitus, hypertension, anemia, and atherosclerosis.^36,42^ It is also important to note that patients with ESKD often develop vascular calcification as part of the CKD-mineral and bone disorder process, as well as due to medications used during dialysis.^43–45^ We propose that pretransplant cardiac assessments are routinely performed for ischemic conditions,^46^ whereas cerebral and peripheral vascular evaluations are often overlooked unless symptoms are present. Without targeted screening or interventions, stroke and peripheral arterial disease may occur earlier than coronary events. Comprehensive vascular assessments, particularly for high-risk patients such as those with diabetes, may help address this gap.^47^ Further studies are needed to guide recommendation.

Transplantation itself is a process of inflammation, starting from the ischemic-reperfusion injury immediately after allograft perfusion and followed by alloimmune recognition and sensitization of the adaptive immune system.^48–50^ In cardiac allograft rejection, inflammation can directly cause atherosclerosis or arteriolosclerosis in coronary arteries, increasing the risk of myocardial infarction and heart failure.^20,51^ However, evidence for the role of kidney allograft rejection in precipitating post-transplant CVEs has mostly depended on kidney allograft dysfunction, which brings atherosclerotic and vascular calcification risks similar to those found in nontransplant CKD patients.^5,10,11,52–56^ Our study found both rejection (TCMR and ABMR) and creatinine clearance to be independent predictors of CVEs. This suggests that rejection is a significant predictor of CVEs on its own, irrespective of its effect on kidney function. Similarly, creatinine clearance reflects an additional, independent pathway influencing CVEs. It is unclear whether kidney allograft rejection leads to further inflammation that propagates systemic endothelial dysfunction or if rejection is merely one of the earlier end points that shares the same pathogenesis as other post-transplant cardiovascular complications. ABMR had a slightly greater effect on CVEs than TCMR, likely due to the broader pathobiology of ABMR, including systemic effects of DSA.

The increased risk of post-transplant CVEs may result not only from rejection pathophysiology, but also from intensified immunosuppressive treatments, such as corticosteroids. In our study, we adjusted for the effect of immunosuppressive medications, including tacrolimus, MPA, and corticosteroid dose at the time of rejection, to assess their effect. We acknowledge that rejection and its treatment are inherently linked as a “package,” making it difficult to fully separate their individual contributions. This is reflected in the multicollinearity observed between rejection and corticosteroid dose in our model. Furthermore, the similarity in rejection treatment protocols among KTRs at our center limits the variability in treatment regimens, restricting our ability to analyze the effects of different doses or variations in rejection treatment. Despite the shared variance between rejection and corticosteroid use, our analysis highlights rejection as a whole as a key driver of post-transplant CVEs. Future studies could use randomized controlled designs or cohorts with varying corticosteroid doses or immunosuppressive agents to better isolate the independent effects of rejection and its treatment.

Hypocalcemia is a recognized risk factor for CVEs and mortality in hemodialysis patients and those who have undergone cardiovascular surgery.^57,58^ The previous study has also shown a 1.3- to 1.8-fold increased mortality risk among KTRs with hypocalcemia,^59^ possibly due to cardiac arrhythmias.^60^ Medications from the pretransplant period are often discontinued and replaced with immunosuppressive drugs. KTRs with postparathyroidectomy hypoparathyroidism or vitamin D deficiency may be at risk for post-transplant hypocalcemia, potentially leading to cardiac complications. Therefore, regular monitoring of serum total calcium or ionized calcium, parathyroid hormone, serum phosphate, and vitamin D status is essential after transplantation. The mechanism underlying the increased incidence of CVEs specifically in KTRs with hypocalcemia remains undefined. However, tacrolimus—a calcineurin inhibitor used in this study—has been associated with hypercalciuria,^61^ which may further predispose KTRs to more severe, unrecognized hypocalcemia. Moreover, since calcineurin function depends on intracellular calcium, patients with hypocalcemia might experience additional suppression of calcineurin activity on top of the inhibition already induced by calcineurin inhibitors. This compounded effect could contribute to dysfunction in other organs, such as skeletal muscle.^62^

Dialysis vintage or preemptive transplantation did not significantly contribute to post-transplant cardiovascular complications in our multivariable model. This may be explained by the inclusion of post-transplant variables, such as diabetes status, HbA1c, and allograft function, which likely have a greater effect than dialysis duration. Previous studies primarily adjusted for pretransplant and perioperative variables,^63–68^ whereas our study accounted for post-transplant factors. In addition, advancements in hemodialysis and hemodiafiltration over recent decades have improved patient outcomes.^69,70^ Studies focusing on patients transplanted before 2010 often reported a strong negative effect of longer dialysis time,^63,65,67,68^ whereas more recent studies show no significant effect on CVEs or cardiovascular death.^71,72^

Our study has several strengths. This is the first comprehensive analysis examining both pretransplant and post-transplant time-updated factors for their effects on post-transplant CVEs. The included KTRs were all Asian, providing valuable information from a real-world study where data have been lacking. The benefits of using competing risk regression analysis over traditional Kaplan-Meier and Cox regression include accounting for the competing events, which improves risk prediction and avoids overestimating of the event probability.^27,32^ Only KTRs receiving tacrolimus with MPA were included, ensuring alignment with current clinical practices and maintaining homogeneity in immunosuppression. We identified kidney allograft rejection as an independent risk factor for post-transplant CVEs, regardless of allograft function. This finding highlights the need for additional cardiovascular screening in patients with ESKD with high immunological risk. Future studies should explore whether enhanced cardiovascular screening can reduce post-transplant CVEs in these patients, irrespective of traditional cardiovascular risk factors. Moreover, we developed a simplified scoring system to predict post-transplant CVEs. This system offers an easier application in clinical practice, potentially improving patient management and outcomes.

This study has several limitations. First, the sample size was smaller than other registries, and while bootstrapping was used for internal validation, external validation is needed. The scoring system, derived from a single cohort, raises concerns about overfitting. We plan to validate it with external cohorts from other Thai transplant centers to assess generalizability. Our cohort, consisting solely of an Asian population, may limit applicability to other ethnicities, and differences in transplant recipient characteristics, such as shorter ischemic times, lower delayed graft function rates, and higher living donor proportions compared with the United States, may affect generalizability. Induction therapy in Thailand predominantly uses IL-2 receptor antagonists (88.1%), similar to practices in South Korea,^73^ but differing from practices in the United States, although the type of induction therapy did not influence CVEs (Supplemental Table 3). In addition, the universal use of statins in our study may not be consistent with practices at other transplant centers. Second, asymptomatic high cardiovascular risk ESKD patients were referred for cardiology evaluation; however, data on coronary interventions were unavailable. Current guidelines suggest that routine coronary interventions are not recommended in asymptomatic transplant candidates,^74,75^ likely minimizing its effect. Third, although BP data were included, information on the effect of antihypertensive classes on CVEs was not available. Our study did not incorporate details on medications that have been shown to slow the progression of CKD—such as sodium-glucose cotransporter 2 inhibitors, glucagon-like peptide 1 agonists, and nonsteroidal mineralocorticoid receptor antagonists—which have demonstrated benefits in preventing CVEs in the nontransplant population. Future studies should include these medications, in addition to traditional agents such as renin-angiotensin-aldosterone system inhibitors, as variables to determine their effect on CVEs in KTRs with rejection. Fourth, post-transplant CMV viral load data were lacking. Although donor-recipient CMV serostatus was not associated with composite outcomes, previous studies suggest that CMV infection increases cardiovascular risk post-transplant.^76^

In conclusion, pretransplant and post-transplant factors influencing CVEs in Thai KTRs were analyzed. Significant factors included recipient age, pretransplant and post-transplant diabetes mellitus, TCMR, ABMR, post-transplant hypocalcemia, HbA1c, and 24-hour urine creatinine clearance. A simplified scoring system was proposed for clinical use. Proper pretransplant screening and post-transplant surveillance in high-risk KTRs may help reduce CVEs.

## Supplementary Material

## Data Availability

All data are included in the manuscript and/or supporting information; Partial restrictions to the data and/or materials apply. The data code supporting the findings of this study are available from the corresponding author upon reasonable request.

## Supplemental Material

Supplemental Table 1. Definitions of the outcome in the study.

Supplemental Table 2. Incidence rate of CVEs at the median time to composite outcomes (3 years post-transplantation).

Supplemental Table 3. Univariable competing-risk regression for composite outcome.

Supplemental Table 4. Bootstrapping for internal validation of the main multivariable model that includes both pretransplant and post-transplant variables.

Supplemental Table 5. Incidence of CVEs by kidney allograft rejection.

Supplemental Table 6. Final multivariable models for the prediction of post-transplant CVEs on the basis of the Akaike information criterion according to each outcome.

Supplemental Table 7. *β*-coefficient in the multivariable models of the simplified score for the prediction of composite post-transplant CVEs.

Supplemental Figure 1. Cumulative incidence function for mortality regarding to the postkidney transplant CVEs (myocardial infarction or ischemia, heart failure, peripheral vascular disease, and ischemic stroke).

Supplemental Figure 2. Trends of post-transplant laboratory parameters (mean±SD), including serum calcium (A), serum phosphate (B), parathyroid hormone (C), serum albumin (D), serum LDL (E), hemoglobin (F), HbA1c (G), systolic BP (H), 24-hour urine creatinine clearance (I), prednisolone dose per day (J), and tacrolimus trough concentration (K).

Supplemental Figure 3. Linearity assessment of age for the risk of composite CVE outcomes.

Supplemental Figure 4. Calibration plot of simplified score using original model as a reference for the prediction of post-transplant CVEs.
