# Supplementary material for: Kidney Allograft Rejection as an Independent Nontraditional Risk Factor for Post-Transplant Cardiovascular Events
Source: Kidney360. 2025 Mar 19;6(7):1176–87. doi: 10.34067/KID.0000000773 (PMC12338360; doi:10.34067/KID.0000000773)
Supplement: Supplementary file 1 [file kidney360-6-1176-s001.pdf]

ASN American Society of Nephrology | JASN Journal of the American Society of Nephrology | KIDNEY360 Accessing Our World From Every Angle | CJASN Clinical Journal of the American Society of Nephrology

ASN Journal Disclosure Form

As per ASN journal policy, I have disclosed any financial relationships or commitments I have held in the past 36 months as included below. I have listed my Current Employer below to indicate there is a relationship requiring disclosure. If no relationship exists, my Current Employer is not listed.

P. Amornkanjanawat has nothing to disclose.

I understand that the information above will be published within the journal article, if accepted, and that failure to comply and/or to accurately and completely report the potential financial conflicts of interest could lead to the following: 1) Prior to publication, article rejection, or 2) Post-publication, sanctions ranging from, but not limited to, issuing a correction, reporting the inaccurate information to the authors' institution, banning authors from submitting work to ASN journals for varying lengths of time, and/or retraction of the published work.

Name: Peemai Amornkanjanawat
Manuscript ID: K360-2024-000918R1
Manuscript Title: Kidney Allograft Rejection as an Independent Non-traditional Risk Factor for Post-transplant Cardiovascular Events
Date of Completion: January 29, 2025
Disclosure Updated Date: January 29, 2025

ASN American Society of Nephrology
JASN Journal of the American Society of Nephrology
KIDNEY360 Accessing Our World From Every Angle
CJASN Clinical Journal of the American Society of Nephrology

ASN Journal Disclosure Form

As per ASN journal policy, I have disclosed any financial relationships or commitments I have held in the past 36 months as included below. I have listed my Current Employer below to indicate there is a relationship requiring disclosure. If no relationship exists, my Current Employer is not listed.

Y. Avihingsanon reports the following:
Employer: King Chulalongkorn Memorial hospital, Chulalongkorn University; Research Funding: Novartis;; Honoraria: Astellas; Advisory or Leadership Role: DMX 201 for FSGS; and Speakers Bureau: Astellas.

I understand that the information above will be published within the journal article, if accepted, and that failure to comply and/or to accurately and completely report the potential financial conflicts of interest could lead to the following: 1) Prior to publication, article rejection, or 2) Post-publication, sanctions ranging from, but not limited to, issuing a correction, reporting the inaccurate information to the authors' institution, banning authors from submitting work to ASN journals for varying lengths of time, and/or retraction of the published work.

Name: Yingyos Avihingsanon
Manuscript ID: K360-2024-000918R2
Manuscript Title: Kidney Allograft Rejection as an Independent Non-traditional Risk Factor for Post-transplant Cardiovascular Events.
Date of Completion: March 4, 2025
Disclosure Updated Date: March 4, 2025

ASN American Society of Nephrology | JASN Journal of the American Society of Nephrology | KIDNEY360 Accessing Our World From Every Angle | CJASN Clinical Journal of the American Society of Nephrology

ASN Journal Disclosure Form

As per ASN journal policy, I have disclosed any financial relationships or commitments I have held in the past 36 months as included below. I have listed my Current Employer below to indicate there is a relationship requiring disclosure. If no relationship exists, my Current Employer is not listed.

S. Eiam-Ong reports the following:
Employer: Chulalongkorn University

I understand that the information above will be published within the journal article, if accepted, and that failure to comply and/or to accurately and completely report the potential financial conflicts of interest could lead to the following: 1) Prior to publication, article rejection, or 2) Post-publication, sanctions ranging from, but not limited to, issuing a correction, reporting the inaccurate information to the authors' institution, banning authors from submitting work to ASN journals for varying lengths of time, and/or retraction of the published work.

Name: Somchai Eiam-Ong
Manuscript ID: K 360-2024-000918R1
Manuscript Title: Kidney allograft rejection as an independent non traditional risk factor for post transplant cardiovascular events
Date of Completion: January 28, 2025
Disclosure Updated Date: May 20, 2024

ASN American Society of Nephrology · JASN Journal of the American Society of Nephrology · KIDNEY360 Accessing Our World From Every Angle · CJASN Clinical Journal of the American Society of Nephrology

ASN Journal Disclosure Form

As per ASN journal policy, I have disclosed any financial relationships or commitments I have held in the past 36 months as included below. I have listed my Current Employer below to indicate there is a relationship requiring disclosure. If no relationship exists, my Current Employer is not listed.

S. Kerr reports the following:
Employer: Faculty of Medicine, Chulalongkorn University and The HIV-Netherlands-Australia-Thailand Research Collaboration; and Honoraria: The Lancet.

I understand that the information above will be published within the journal article, if accepted, and that failure to comply and/or to accurately and completely report the potential financial conflicts of interest could lead to the following: 1) Prior to publication, article rejection, or 2) Post-publication, sanctions ranging from, but not limited to, issuing a correction, reporting the inaccurate information to the authors' institution, banning authors from submitting work to ASN journals for varying lengths of time, and/or retraction of the published work.

Name: Stephen J. Kerr
Manuscript ID: K360-2024-000918R2
Manuscript Title: Kidney Allograft Rejection as an Independent Non-traditional Risk Factor for Post-transplant Cardiovascular Events
Date of Completion: March 4, 2025
Disclosure Updated Date: January 27, 2025

ASN American Society of Nephrology | JASN Journal of the American Society of Nephrology | KIDNEY360 Accessing Our World From Every Angle | CJASN Clinical Journal of the American Society of Nephrology

ASN Journal Disclosure Form

As per ASN journal policy, I have disclosed any financial relationships or commitments I have held in the past 36 months as included below. I have listed my Current Employer below to indicate there is a relationship requiring disclosure. If no relationship exists, my Current Employer is not listed.

A. Leelahavanichkul reports the following:
Employer: Chulalongkorn University

I understand that the information above will be published within the journal article, if accepted, and that failure to comply and/or to accurately and completely report the potential financial conflicts of interest could lead to the following: 1) Prior to publication, article rejection, or 2) Post-publication, sanctions ranging from, but not limited to, issuing a correction, reporting the inaccurate information to the authors' institution, banning authors from submitting work to ASN journals for varying lengths of time, and/or retraction of the published work.

Name: Asada Leelahavanichkul
Manuscript ID: K360-2024-000918R1
Manuscript Title: Kidney Allograft Rejection as an Independent Non-traditional Risk Factor for Post-transplant Cardiovascular Events
Date of Completion: January 27, 2025
Disclosure Updated Date: January 27, 2025

ASN American Society of Nephrology | JASN Journal of the American Society of Nephrology | KIDNEY360 Accessing Our World From Every Angle | CJASN Clinical Journal of the American Society of Nephrology

ASN Journal Disclosure Form

As per ASN journal policy, I have disclosed any financial relationships or commitments I have held in the past 36 months as included below. I have listed my Current Employer below to indicate there is a relationship requiring disclosure. If no relationship exists, my Current Employer is not listed.

K. Praditpornsilpa reports the following:
Employer: Chulalongkorn University; Research Funding: Fresenius Kabi; and Speakers Bureau: Fresenius Kabi.

I understand that the information above will be published within the journal article, if accepted, and that failure to comply and/or to accurately and completely report the potential financial conflicts of interest could lead to the following: 1) Prior to publication, article rejection, or 2) Post-publication, sanctions ranging from, but not limited to, issuing a correction, reporting the inaccurate information to the authors' institution, banning authors from submitting work to ASN journals for varying lengths of time, and/or retraction of the published work.

Name: Kearkiat Praditpornsilpa
Manuscript ID: K360-2024-000918R2
Manuscript Title: Kidney Allograft Rejection as an Independent Non-traditional Risk Factor for Post-transplant Cardiovascular Events
Date of Completion: March 6, 2025
Disclosure Updated Date: March 6, 2025

ASN American Society of Nephrology | JASN Journal of the American Society of Nephrology | KIDNEY360 Accessing Our World From Every Angle | CJASN Clinical Journal of the American Society of Nephrology

ASN Journal Disclosure Form

As per ASN journal policy, I have disclosed any financial relationships or commitments I have held in the past 36 months as included below. I have listed my Current Employer below to indicate there is a relationship requiring disclosure. If no relationship exists, my Current Employer is not listed.

P. Tantiyavarong reports the following:
Employer: Thammasat University Hospital; and Honoraria: DKSH (Thailand).

I understand that the information above will be published within the journal article, if accepted, and that failure to comply and/or to accurately and completely report the potential financial conflicts of interest could lead to the following: 1) Prior to publication, article rejection, or 2) Post-publication, sanctions ranging from, but not limited to, issuing a correction, reporting the inaccurate information to the authors' institution, banning authors from submitting work to ASN journals for varying lengths of time, and/or retraction of the published work.

Name: Pichaya Tantiyavarong
Manuscript ID: K360-2024-000918R1
Manuscript Title: Kidney Allograft Rejection as an Independent Non-traditional Risk Factor for Post-transplant Cardiovascular Events
Date of Completion: January 28, 2025
Disclosure Updated Date: January 26, 2025

ASN American Society of Nephrology | JASN Journal of the American Society of Nephrology | KIDNEY360 Accessing Our World From Every Angle | CJASN Clinical Journal of the American Society of Nephrology

ASN Journal Disclosure Form

As per ASN journal policy, I have disclosed any financial relationships or commitments I have held in the past 36 months as included below. I have listed my Current Employer below to indicate there is a relationship requiring disclosure. If no relationship exists, my Current Employer is not listed.

N. Townamchai reports the following:
Employer: Division of Nephrology, Chulalongkorn University; and Honoraria: Pfizer; AstraZaneca; Boehringer; Astellas.

I understand that the information above will be published within the journal article, if accepted, and that failure to comply and/or to accurately and completely report the potential financial conflicts of interest could lead to the following: 1) Prior to publication, article rejection, or 2) Post-publication, sanctions ranging from, but not limited to, issuing a correction, reporting the inaccurate information to the authors' institution, banning authors from submitting work to ASN journals for varying lengths of time, and/or retraction of the published work.

Name: Natavudh Townamchai
Manuscript ID: K360-2024-000918R1
Manuscript Title: Kidney Allograft Rejection as an Independent Non-traditional Risk Factor for Post-transplant Cardiovascular Events
Date of Completion: January 28, 2025
Disclosure Updated Date: May 20, 2024

ASN American Society of Nephrology | JASN Journal of the American Society of Nephrology | KIDNEY360 Accessing Our World From Every Angle | CJASN Clinical Journal of the American Society of Nephrology

ASN Journal Disclosure Form

As per ASN journal policy, I have disclosed any financial relationships or commitments I have held in the past 36 months as included below. I have listed my Current Employer below to indicate there is a relationship requiring disclosure. If no relationship exists, my Current Employer is not listed.

S. Udomkarnjananun has nothing to disclose.

I understand that the information above will be published within the journal article, if accepted, and that failure to comply and/or to accurately and completely report the potential financial conflicts of interest could lead to the following: 1) Prior to publication, article rejection, or 2) Post-publication, sanctions ranging from, but not limited to, issuing a correction, reporting the inaccurate information to the authors' institution, banning authors from submitting work to ASN journals for varying lengths of time, and/or retraction of the published work.

Name: Suwasin Udomkarnjananun
Manuscript ID: K360-2024-000918R1
Manuscript Title: Kidney Allograft Rejection as an Independent Non-traditional Risk Factor for Post-transplant Cardiovascular Events
Date of Completion: January 24, 2025
Disclosure Updated Date: May 17, 2024

ASN American Society of Nephrology | JASN Journal of the American Society of Nephrology | KIDNEY360 Accessing Our World From Every Angle | CJASN Clinical Journal of the American Society of Nephrology

ASN Journal Disclosure Form

As per ASN journal policy, I have disclosed any financial relationships or commitments I have held in the past 36 months as included below. I have listed my Current Employer below to indicate there is a relationship requiring disclosure. If no relationship exists, my Current Employer is not listed.

T. Wuttiputhanun has nothing to disclose.

I understand that the information above will be published within the journal article, if accepted, and that failure to comply and/or to accurately and completely report the potential financial conflicts of interest could lead to the following: 1) Prior to publication, article rejection, or 2) Post-publication, sanctions ranging from, but not limited to, issuing a correction, reporting the inaccurate information to the authors' institution, banning authors from submitting work to ASN journals for varying lengths of time, and/or retraction of the published work.

Name: Thunyatorn Wuttiputhanun
Manuscript ID: K360-2024-000918R1
Manuscript Title: Kidney Allograft Rejection as an Independent Non-traditional Risk Factor for Post-transplant Cardiovascular Events
Date of Completion: January 28, 2025
Disclosure Updated Date: January 28, 2025
