# Supplementary material for: Kidney Allograft Rejection as an Independent Nontraditional Risk Factor for Post-Transplant Cardiovascular Events
Source: Kidney360. 2025 Mar 19;6(7):1176–87. doi: 10.34067/KID.0000000773 (PMC12338360; doi:10.34067/KID.0000000773)

SUPPLEMENTAL MATERIALS

Supplemental Table 1: Definitions of the outcome in the study. These definitions were consistently applied to all patients in the study, and every diagnosis was confirmed after thorough chart review to ensure accuracy and consistency with the clinical and investigational standards

Outcomes	Definitions
Myocardial infarction (MI) and/or ischemia	MI was diagnosed based on standard clinical criteria, which include a combination of clinical presentation (e.g., chest pain), electrocardiographic (ECG) changes (e.g., ST-segment elevation or depression), and elevated cardiac biomarkers (e.g., troponin levels). For ischemia, defined as a lack of blood flow to the heart muscle, the diagnosis was made through clinical symptoms (e.g., chest pain, shortness of breath) and confirmed with non-invasive tests such as stress testing (e.g., exercise treadmill test or pharmacologic stress) or coronary angiography, showing evidence of coronary artery disease but without elevated cardiac biomarkers. Both overt and silent MIs were included, with confirmation through objective criteria, such as elevated cardiac biomarkers (e.g., troponin levels), ECG changes, and/or imaging findings.
Ischemic stroke	The diagnosis of ischemic stroke was made according to clinical presentation and neuroimaging findings. All patients with suspected ischemic stroke underwent brain imaging, typically via computed tomography (CT) or magnetic resonance imaging (MRI), to confirm the presence of an ischemic lesion. Stroke was classified as ischemic if the lesion was due to an embolic or thrombotic event, and the diagnosis was confirmed through a detailed review of the clinical and radiological findings.
Heart failure (HF)	HF included both heart failure with reduced ejection fraction (HFrEF) and heart failure with preserved ejection fraction (HFpEF). The diagnosis was made based on clinical criteria such as symptoms (e.g., dyspnea, fatigue, and edema) and diagnostic tests, primarily echocardiography to assess left ventricular ejection fraction (LVEF). Both HFrEF and HFpEF were included, and the diagnosis was confirmed through chart review.
Peripheral arterial disease (PAD)	PAD was diagnosed based on clinical presentation and confirmatory diagnostic tests. Patients presenting with symptoms such as claudication, non-healing ulcers, or other signs of ischemia were evaluated using non-invasive methods like ankle-brachial index (ABI), duplex ultrasonography, or contrast angiography if necessary. Diagnosis was confirmed through these diagnostic tools.

Cardiovascular death	Cardiovascular death was defined as death due to a cardiovascular event, including heart failure, myocardial infarction, ischemic stroke, or other cardiovascular causes, as confirmed through death certificates and medical records.

Supplemental Table 2: Incidence rate of cardiovascular events at the median time to composite outcomes (3 years post-transplantation).

Parameters	The first 3 years post-transplantation	After 3 years post-transplantation
**Incidence rate of composite outcome** (per 1000 patient-years, 95%CI)	19.2 (13.0 – 28.1)	13.7 (9.5 – 19.9)
**All-cause mortality** (per 1000 patient-years, 95%CI)	7.9 (4.4 – 14.3)	9.7 (6.3 – 14.8)
**Events**		
**Myocardial infarction or ischemia** (per 1000 patient-years, 95%CI)	5.1 (2.4 – 10.6)	8.5 (5.4 – 13.5)
**Heart failure** (per 1000 patient-years, 95%CI)	6.5 (3.4 – 12.6)	4.7 (2.5 – 8.7)
**Peripheral Arterial disease** (per 1000 patient-years, 95%CI)	6.5 (3.4 – 12.5)	3.3 (1.6 – 6.9)
**Ischemic stroke** (per 1000 patient-years, 95%CI)	4.3 (1.9 – 9.6)	2.8 (1.3 – 6.2)
**Death from cardiovascular disease** (per 1000 patient-years, 95%CI)	2.2 (0.7 – 6.7)	2.8 (1.2 – 6.1)

Supplemental Table 3: Univariable competing-risk regression for composite outcome.

Variables	Univariable analysis		
	SHR	95%CI	p-value
**Recipient characteristics**			
Recipient age	1.06	1.04 – 1.09	<0.001
Recipient male sex	0.98	0.57 – 1.68	0.940
Body mass index	1.13	1.06 – 1.20	<0.001
Glomerular disease as a cause of native kidney disease	0.57	0.29 – 1.14	0.112
Preemptive transplantation	0.69	0.16 – 2.92	0.615
Dialysis vintage	1.06	0.99 – 1.15	0.077
Secondary kidney transplantation	3.16	1.27 – 7.85	0.013
Pre-transplant diabetes mellitus	8.12	4.74 – 13.92	<0.001
Cardiovascular disease^{+}	3.94	2.13 – 7.30	<0.001
Antiplatelet uses^{+}	3.43	0.57 – 7.99	0.093
History of malignancy	1.09	0.93 – 1.27	0.290
History of parathyroidectomy	1.23	0.45 – 3.36	0.688
Smoking history	1.22	0.58 – 2.57	0.604
**Transplant characteristics**			
Donor age	1.02	0.99 – 1.05	0.060
Donor male sex	1.06	0.61 – 1.85	0.709
Deceased donor	1.97	1.10 – 3.53	0.023
Donor terminal creatinine	1.05	0.90 – 1.22	0.546
Donor hypertension	2.20	0.93 – 5.19	0.072
Donor diabetes mellitus	3.92	0.58 – 26.60	0.163

ABO incompatibility	0.89	0.27 – 2.88	0.846
Total ischemic time	1.00	0.99 – 1.00	0.255
Number of human leukocyte mismatch	1.09	0.92 – 1.30	0.270
Panel reactive antibody	1.00	0.99 – 1.01	0.403
Anti-thymocyte globulin induction (compared to IL2 receptor antagonists)	1.58	0.80 – 3.11	0.185
Delayed graft function	2.35	1.34 – 4.12	0.003
Length of stay during the first admission	1.03	1.01 – 1.04	0.002
CMV D+/R- (compared to CMV D+/R+)	0.43	0.15 – 1.22	0.112
Multiorgan transplantation	2.10	0.51 – 3.74	0.142
**Post-transplant variables***			
Newly diagnosed post-transplant diabetes mellitus	2.15	1.03 – 4.49	0.041
Previous T-cell mediated rejection	5.94	2.94 – 12.01	<0.001
Previous Antibody-mediated rejection	2.66	1.04 – 6.82	0.041
Systolic blood pressure	1.03	1.01 – 1.05	0.002
Diastolic blood pressure	0.98	0.95 – 1.01	0.164
Hemoglobin level	0.83	0.72 – 0.96	0.012
Serum sodium	0.99	0.87 – 1.12	0.840
Serum potassium	1.40	0.82 – 2.40	0.222
Serum bicarbonate	0.98	0.87 – 1.11	0.794
Serum calcium	0.57	0.38 – 0.85	0.005
Serum phosphate	1.04	0.67 – 1.60	0.871
Serum albumin	0.31	0.19 – 0.50	<0.001
Serum high-density lipoprotein	0.99	0.97 – 1.00	0.161

Serum low-density lipoprotein	0.99	0.99 – 1.01	0.661
Serum parathyroid hormone	1.00	0.99 – 1.00	0.378
Serum alkaline phosphatase	0.99	0.99 – 1.00	0.869
Serum uric acid	1.03	0.87 – 1.23	0.704
Serum creatinine[#]	1.16	0.96 – 1.34	0.081
Fasting plasma glucose	1.01	1.00 – 1.01	<0.001
Hemoglobin A1c	1.29	1.17 – 1.43	<0.001
24-hour urine protein	1.20	1.04 – 1.36	0.012
24-hour urine creatinine clearance[#]	0.97	0.96 – 0.99	<0.001
Tacrolimus concentration	1.07	0.97 – 1.19	0.186
Mycophenolic acid dose per day	0.88	0.77 - 1.01	0.060
Prednisolone dose	1.06	1.04 – 1.07	<0.001

*Time-varying variables during the post-transplant follow-up

[+] Both antiplatelet use and previous history of cardiovascular disease were candidates for inclusion in the multivariable model based on a p-value of less than 0.1. However, only previous history of cardiovascular disease was included in the multivariable model due to its stronger association (based on its p-value) and its collinearity with antiplatelet use.

[#]Both serum creatinine and 24-hour urine creatinine clearance were candidates for inclusion in the multivariable model based on a p-value of less than 0.1. However, only 24-hour urine creatinine clearance was included due to its stronger association with the outcome and to avoid collinearity between the two variables.

SHR; sub-hazard ratio

Supplemental Table 4: Bootstrapping for internal validation of the main multivariable model that includes both pre- and post-transplant variables.

Variables	Original SHR	Bootstrap Mean Estimate	Standard error	95% confidence interval
Recipient age	1.048	1.050	0.018	1.015-1.085
Recipient diabetes mellitus	6.081	6.185	2.516	2.787-13.729
Newly diagnosed post-transplant diabetes mellitus	2.698	2.720	1.541	0.896-8.256
T cell-mediated rejection	3.004	2.937	1.612	1.001-8.612
Antibody-mediated rejection	3.381	3.344	2.211	1.542-10.211
Post-transplant calcium concentrations	0.594	0.583	0.153	0.348-0.976
Post-transplant HbA1c	1.155	1.146	0.111	0.947-1.386
24-hour urine creatinine clearance	0.978	0.978	0.007	0.966-0.991

Supplemental Table 5: Incidence of cardiovascular events by kidney allograft rejection.

**Parameters**	**KTR with allograft rejection**	**KTR without allograft rejection**
**Overall follow-up time**, (median, IQR), y	6.2 (4.0 – 8.6)	6.1 (3.7 – 9.0)
**Time to composite outcome**, (median, IQR), y	2.0 (1.1 – 5.2)	3.6 (1.2 – 6.6)
**Incidence rate of composite outcome** (per 1000 patient-years, 95%CI)	60.9 (36.1 – 102.8)	13.1 (9.6 – 17.9)
**All-cause mortality** (per 1000 patient-years, 95%CI)	17.7 (7.4 – 42.6)	8.4 (5.7 – 12.4)
**Events**		
**Myocardial infarction or ischemia** (per 1000 patient-years, 95%CI)	39.2 (21.1 – 72.8)	4.6 (2.7 – 7.8)
**Heart failure** (per 1000 patient-years, 95%CI)	26.6 (12.7 – 55.7)	3.9 (2.2 – 6.9)
**Peripheral Arterial disease** (per 1000 patient-years, 95%CI)	11.2 (3.6 – 34.7)	4.3 (2.5 – 7.3)
**Ischemic stroke** (per 1000 patient-years, 95%CI)	11.0 (3.6 – 34.2)	2.9 (1.5 – 5.6)
**Death from cardiovascular disease** (per 1000 patient-years, 95%CI)	10.6 (3.4 – 33.0)	1.9 (0.9 – 4.3)

Supplemental Table 6: Final multivariable models for the prediction of post-transplant cardiovascular events based on the Akaike information criterion according to each outcome. The low number of events for each separate outcome limits the accuracy and generalizability of these models compared to the primary composite outcome model, which was based on a more robust number of events.

**Cardiovascular death (9 events, C-statistic 0.79)**	**SHR**	**95%CI**	**P-value**
Pre-transplant recipient diabetes mellitus	4.83	1.24 – 18.94	0.024
Previous T-cell mediated rejection	1.56	1.27 – 3.19	0.016
Previous Antibody-mediated rejection	4.31	2.67 – 6.98	<0.001
Post-transplant HbA1c	1.28	0.97 – 1.68	0.078
Post-transplant 24-hour urine creatinine clearance	0.97	0.95 – 0.99	0.001
**Myocardial infarction/ischemia (25 events, C-statistic 0.88)**	**SHR**	**95%CI**	**P-value**
Recipient age	1.07	1.03 – 1.10	<0.001
Pre-transplant recipient diabetes mellitus	6.03	2.25 – 16.15	<0.001
Newly diagnosed post-transplant diabetes mellitus	3.56	1.10 – 11.53	0.034
Previous T-cell mediated rejection	7.11	2.46 – 20.51	<0.001
Previous Antibody-mediated rejection	4.04	1.36 – 11.95	0.012
**Heart failure (19 events, C-statistic 0.82)**	**SHR**	**95%CI**	**P-value**
Recipient age	1.10	1.03 – 1.17	0.003

Recipient body mass index	1.21	1.07 – 1.37	0.003
Previous T-cell mediated rejection	7.31	1.74 – 30.74	0.007
Post-transplant calcium concentrations	0.38	0.15 – 0.97	0.044
Post-transplant 24-hour urine creatinine clearance	0.96	0.94 – 0.99	0.003
**Peripheral arterial disease (16 events, C-statistic 0.82)**	**SHR**	**95%CI**	**P-value**
Pre-transplant recipient diabetes mellitus	8.29	3.73 – 18.41	<0.001
Newly diagnosed post-transplant diabetes mellitus	3.54	1.29 – 6.44	<0.001
Post-transplant systolic blood pressure	1.03	1.01 – 1.05	0.049
Post-transplant HbA1c	1.58	1.25 – 2.00	<0.001
Post-transplant 24-hour urine creatinine clearance	0.96	0.94 – 0.98	0.001
**Ischemic stroke (12 events, C-statistic 0.78)**	**SHR**	**95%CI**	**P-value**
Pre-transplant recipient diabetes mellitus	4.58	1.20 – 17.56	0.026
Previous T-cell mediated rejection	4.61	3.25 – 6.53	<0.001
Previous Antibody-mediated rejection	6.37	1.54 – 26.37	0.011
Post-transplant 24-hour urine protein	1.26	0.89 – 1.78	0.192

Supplemental Table 7: Beta-coefficient in the multivariable models of the simplified score for the prediction of composite post-transplant cardiovascular events.

**Variables**	**Multivariable analysis of simplified score**		
	**Beta-coefficient**	**95%CI**	**P-value**
Recipient age ≥ 40 years at transplantation	3.296	0.897 to 5.693	0.007
Pre-transplant recipient diabetes mellitus	1.843	1.107 to 2.578	<0.001
Newly diagnosed post-transplant diabetes mellitus	0.852	-0.108 to 1.812	0.082
Previous T-cell mediated rejection	1.287	0.456 to 2.120	0.004
Previous Antibody-mediated rejection	1.883	0.910 to 2.994	0.001
Post-transplant calcium concentrations < 8.5 mg/dL	1.057	0.345 to 1.769	0.004
Post-transplant HbA1c ≥ 7	0.685	-0.088 to 1.058	0.097
24-hour urine creatinine clearance < 60 mL/min	0.826	0.152 to 1.499	0.016

Supplemental Figure 1: Cumulative incidence function for mortality regarding to the post-kidney transplant cardiovascular events (myocardial infarction or ischemia, heart failure, peripheral vascular disease, and ischemic stroke).

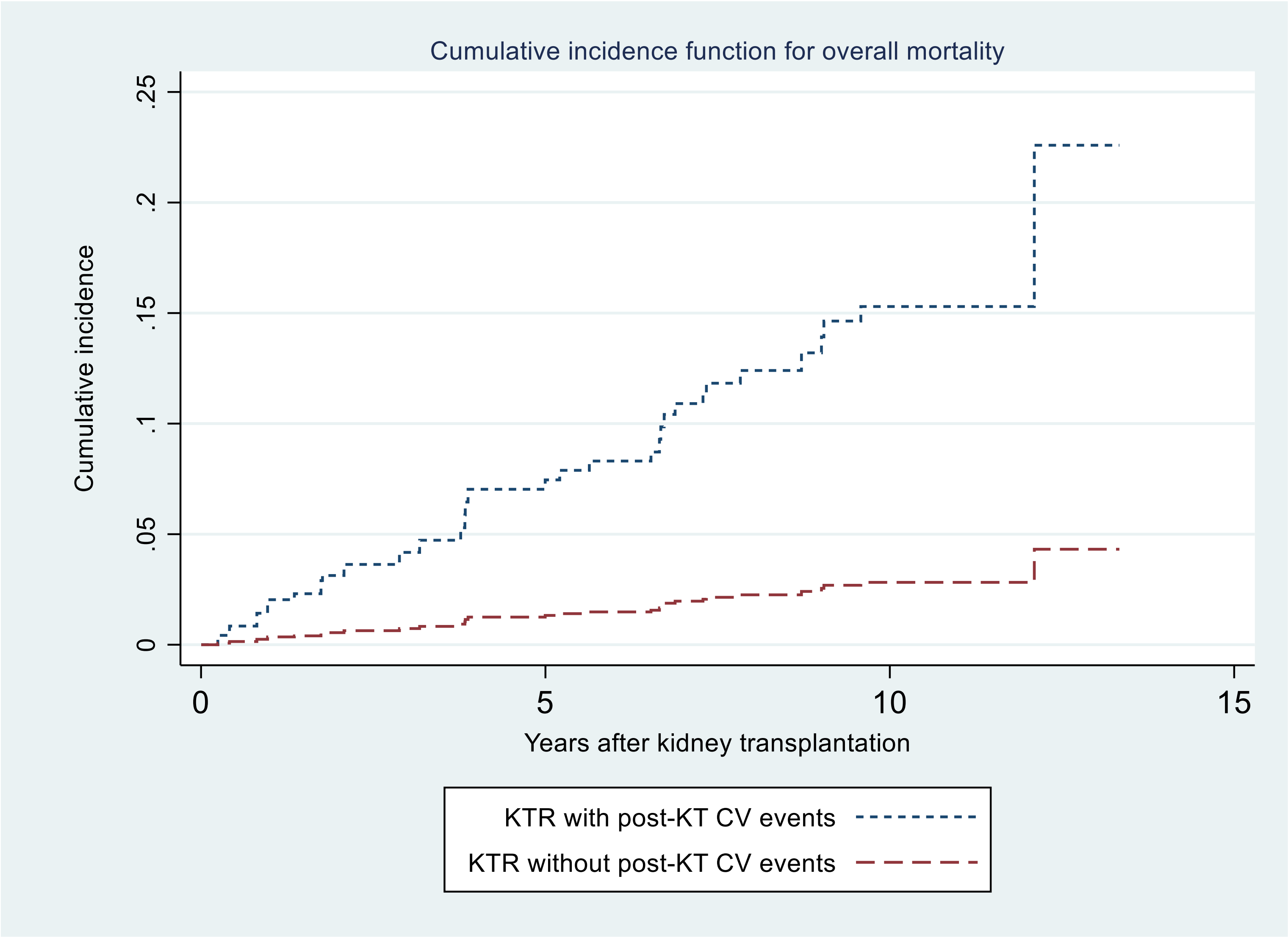

Supplemental Figure 2: Trends of post-transplant laboratory parameters (mean ± SD), including serum calcium (A), serum phosphate (B), parathyroid hormone (C), serum albumin (D), serum low-density lipoprotein (E), hemoglobin (F), HbA1c (G), systolic blood pressure (H), 24-hour urine creatinine clearance (I), prednisolone dose per day (J), tacrolimus trough concentration in median and IQR (K), and mycophenolic acid dose per day (equivalent to MMF) (L).

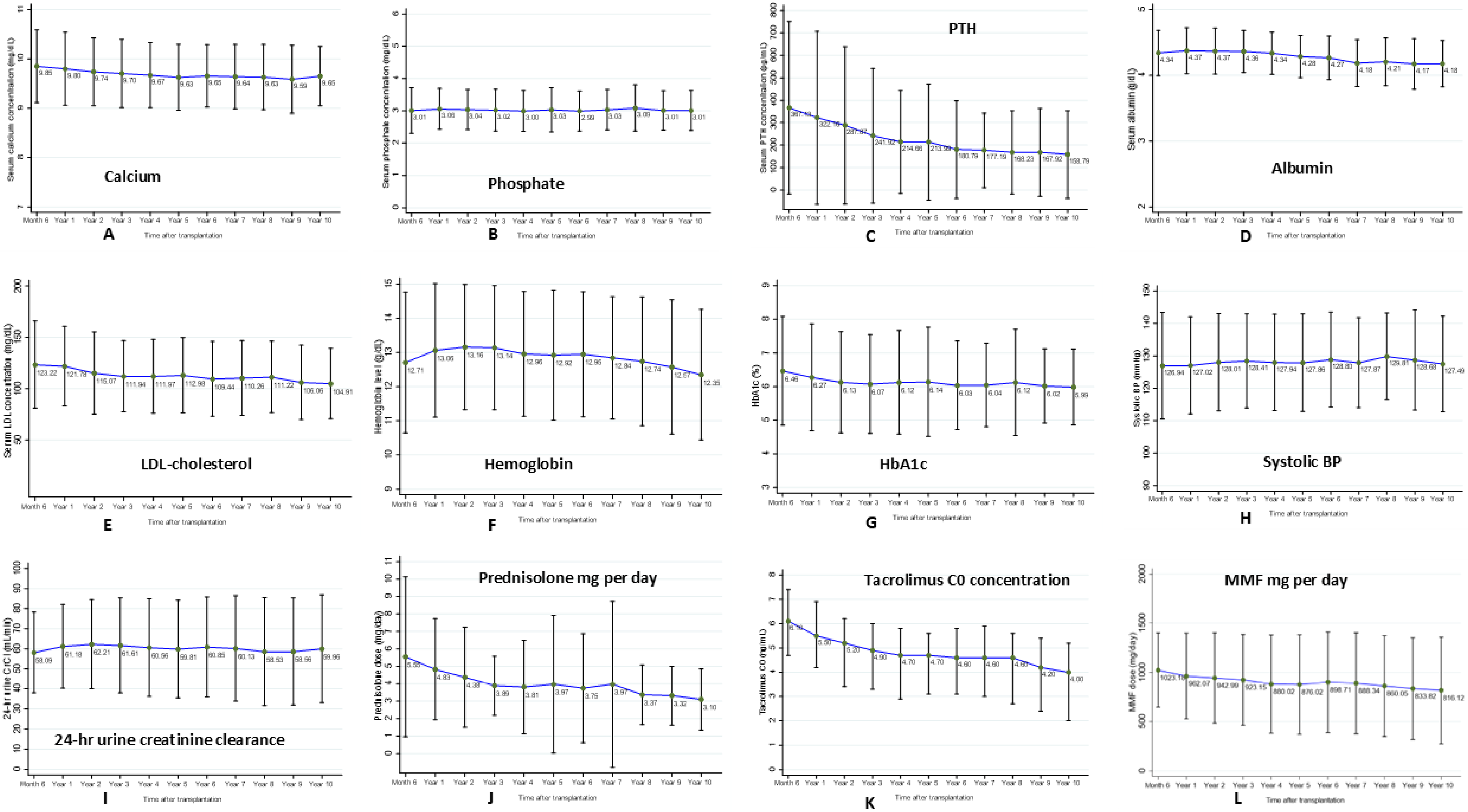

Supplemental Figure 3: Linearity assessment of age for the risk of composite cardiovascular event outcomes.

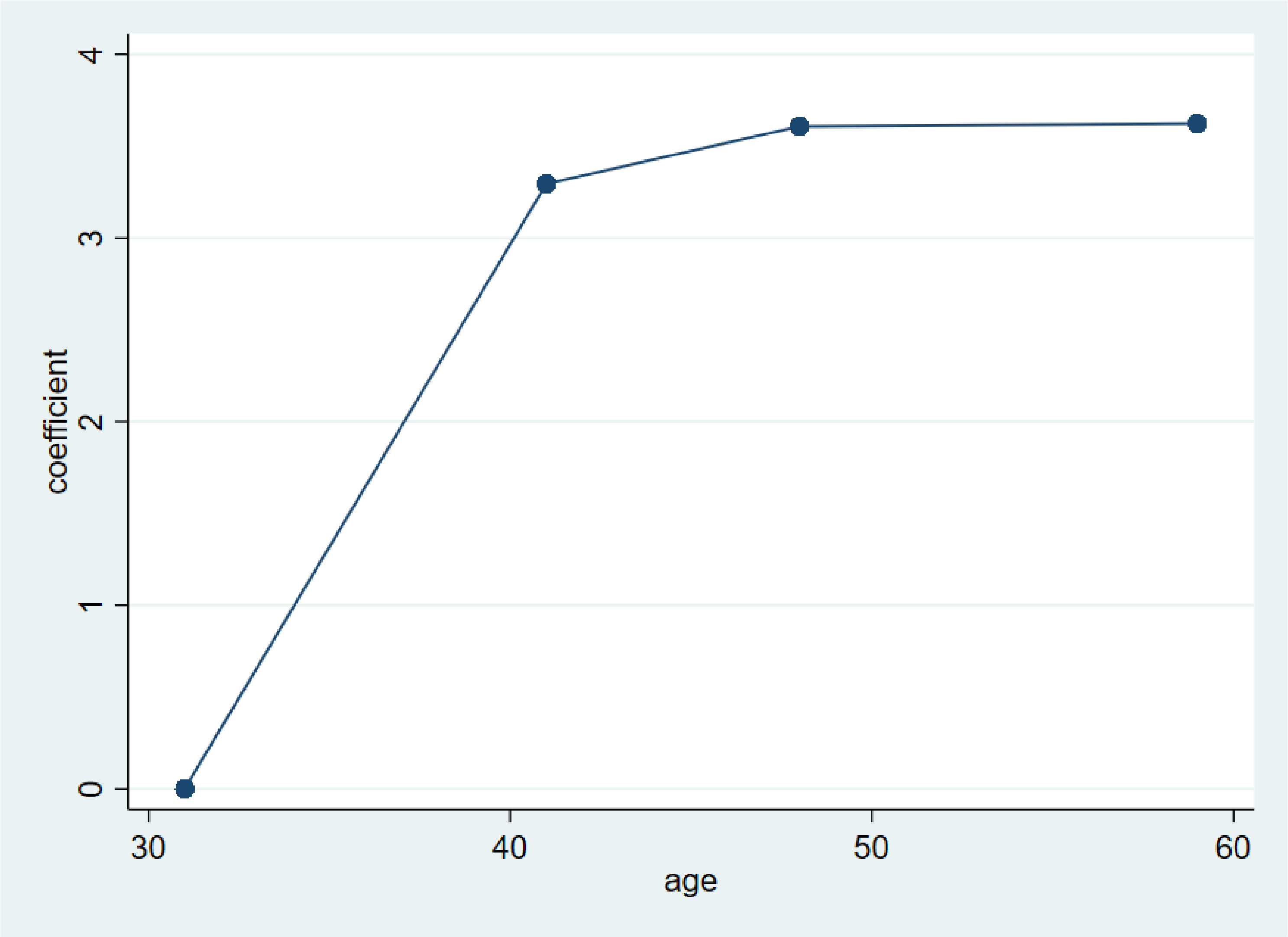

Supplemental Figure 4: Calibration plot of simplified score using original model as a reference for the prediction of post-transplant cardiovascular events.

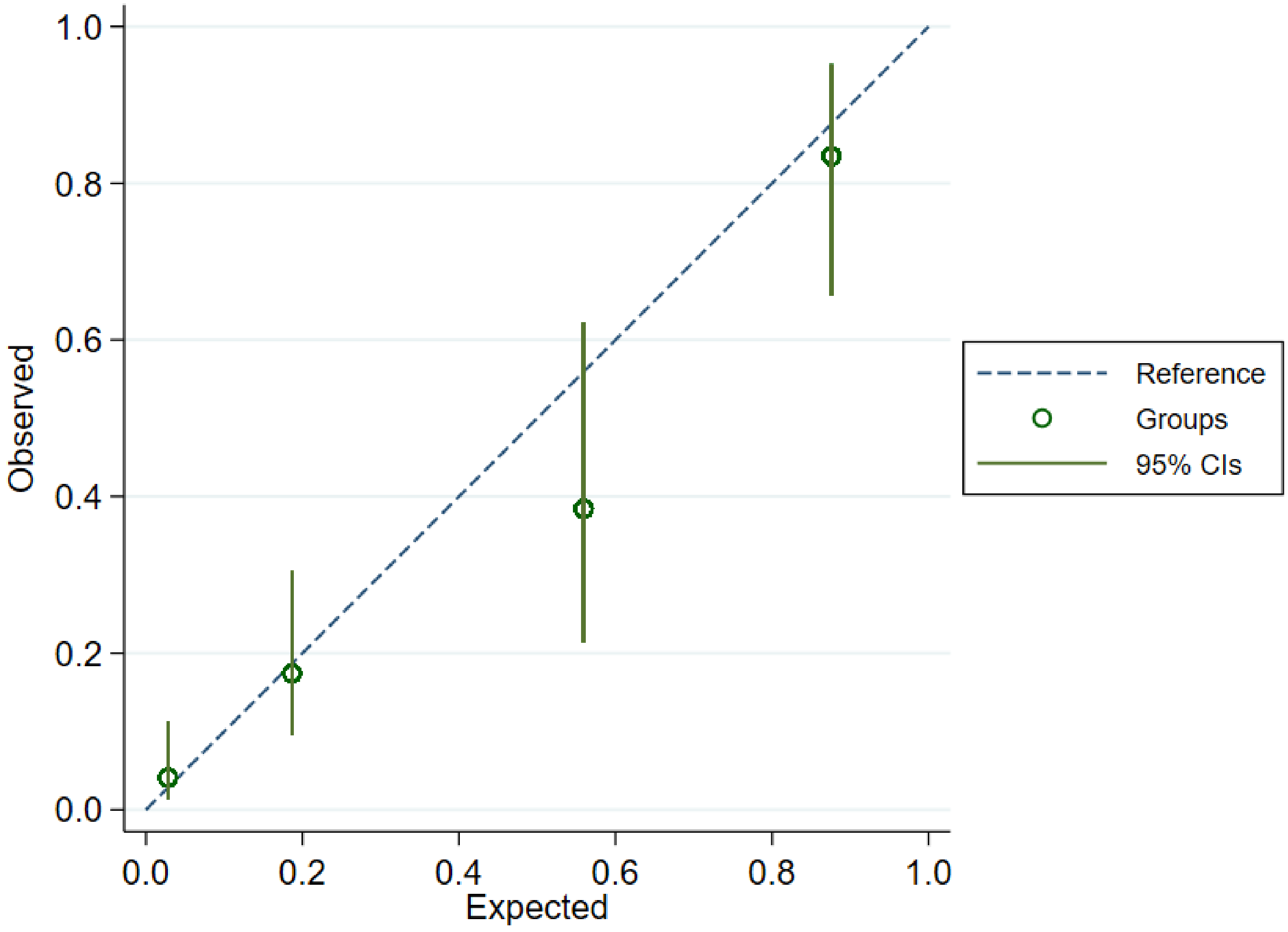

Supplement: Supplementary file 2 [file kidney360-6-1176-s002.pdf]
